# Seasonal patterns in reproductive success of temperate‐breeding birds: Experimental tests of the date and quality hypotheses

**DOI:** 10.1002/ece3.2815

**Published:** 2017-02-28

**Authors:** Vanessa B. Harriman, Russell D. Dawson, Lauren E. Bortolotti, Robert G. Clark

**Affiliations:** ^1^ Department of BiologyUniversity of SaskatchewanSaskatoonSKCanada; ^2^ Ecosystem Science and ManagementUniversity of Northern British ColumbiaPrince GeorgeBCCanada; ^3^ Environment and Climate Change CanadaPrairie and Northern Wildlife Research CentreSaskatoonSKCanada; ^4^ Present address: Government of ManitobaWildlife and Fisheries BranchWinnipegMBCanada; ^5^ Present address: Institute for Wetland and Waterfowl ResearchDucks Unlimited CanadaStonewallMBCanada

**Keywords:** food supply, hatching date, offspring quality, parental quality, *Tachycineta bicolor*, tree swallow

## Abstract

For organisms in seasonal environments, individuals that breed earlier in the season regularly attain higher fitness than their late‐breeding counterparts. Two primary hypotheses have been proposed to explain these patterns: The quality hypothesis contends that early breeders are of better phenotypic quality or breed on higher quality territories, whereas the date hypothesis predicts that seasonally declining reproductive success is a response to a seasonal deterioration in environmental quality. In birds, food availability is thought to drive deteriorating environmental conditions, but few experimental studies have demonstrated its importance while also controlling for parental quality. We tested predictions of the date hypothesis in tree swallows (*Tachycineta bicolor*) over two breeding seasons and in two locations within their breeding range in Canada. Nests were paired by clutch initiation date to control for parental quality, and we delayed the hatching date of one nest within each pair. Subsequently, brood sizes were manipulated to mimic changes in per capita food abundance, and we examined the effects of manipulations, as well as indices of environmental and parental quality, on nestling quality, fledging success, and return rates. Reduced reproductive success of late‐breeding individuals was causally related to a seasonal decline in environmental quality. Declining insect biomass and enlarged brood sizes resulted in nestlings that were lighter, in poorer body condition, structurally smaller, had shorter and slower growing flight feathers and were less likely to survive to fledge. Our results provide evidence for the importance of food resources in mediating seasonal declines in offspring quality and survival.

## 1 Introduction

A decline in fitness‐related traits among individuals that reproduce later in the season has been observed in diverse plant and animal taxa (Anderson, Faulds, Atlas, Pess, & Quinn, 2010; Kelly & Levin, 2000; Uller & Olsson, 2010; Varpe, Jørgensen, Tarling, & Fiksen, 2007). Reduced reproductive success with later nesting dates has been particularly well documented in temperate‐breeding birds. Declining clutch size (Öberg, Pärt, Arlt, Laugen, & Low, 2013; Winkler et al., 2014), offspring quality (Dubiec & Cichoń, 2001; Siikamäki, 1998), survival (Gurney, Clark, & Slattery, 2012; Öberg et al., 2013; Shutler, Clark, Fehr, & Diamond, 2006), and longevity (Saino et al., 2012) have all been reported. Decisions about timing of breeding have fitness consequences (Daan & Tinbergen, 1997) that are likely mediated by individual and environmental quality (Reed et al., 2009), these being the two primary hypotheses proposed to explain seasonal variation in reproductive success.

The quality hypothesis asserts that early‐breeding individuals are of better phenotypic quality and/or breed on higher quality territories than late‐breeding individuals (Price, Kirkpatrick, & Arnold, 1988; Verhulst & Tinbergen, 1991). The date hypothesis states that seasonally declining reproductive success is a response to a seasonal deterioration in environmental quality (Perrins, 1970; Verhulst & Nilsson, 2008). The two hypotheses are not mutually exclusive, and their effects may be manifested simultaneously, at different times throughout the breeding season or at different life‐history stages (Grüebler & Naef‐Daenzer, 2010; Gurney et al., 2012; Verhulst, Vanbalen, & Tinbergen, 1995). Identifying when and how environmental quality mediates reproductive success is particularly important in making predictions about environmental effects on avian populations under changing climatic conditions (Lyon, Chaine, & Winkler, 2008; Verhulst & Nilsson, 2008).

Mechanisms underlying a seasonal decline in environmental quality may be complex; however, food availability is often proposed as the most important factor influencing seasonal variation in reproductive success of many avian species (Perrins, 1970; Siikamäki, 1998; Verboven, Tinbergen, & Verhulst, 2001). It has generally been assumed that birds time breeding so that the nestling period corresponds with periods of peak food availability, and reproductive success has been frequently linked with seasonal declines in food abundance both in observational (Norris, 1993; Öberg et al., 2013) and experimental studies (Burger et al., 2012; Verboven et al., 2001). Nonetheless, we are aware of only one experimental investigation testing the assertion that food abundance mediates seasonally declining reproductive success while controlling for parental quality (Siikamäki, 1998). This is likely due to the challenge of performing a manipulation that forces parents to raise their offspring during an unintended period of time (i.e., with different food supplies) without altering parental quality (Verhulst & Nilsson, 2008).

We conducted a series of manipulations to test predictions associated with the date hypothesis, while accounting for parental quality, in tree swallows (*Tachycineta bicolor*) over two breeding seasons and in two locations within their breeding range in Canada. Apparent recruitment rates of tree swallows are negatively related to hatching date (Shutler et al., 2006) and robust tests of this hypothesis are absent among North American aerial insectivores, a guild of birds experiencing population declines in many regions of this continent (Nebel, Mills, McCracken, & Taylor, 2010; Shutler et al., 2012). To account for potential effects of parental quality, we paired nests by clutch initiation date and delayed the hatching of one nest within the pair. We then manipulated brood size to mimic changes in per capita food abundance (Bortolotti, Harriman, Clark, & Dawson, 2011; Shutler et al., 2006). We examined the effects of manipulations, as well as indices of environmental (e.g., insect biomass) and parental (e.g., body condition) quality, on nestling quality, fledging success, and return rates. If seasonally declining reproductive success is attributable to seasonally declining food abundance, we predicted that (1) nestlings in enlarged broods (i.e., with reduced per capita food) would be of lower quality and less likely to survive to fledging or return in subsequent years than their control counterparts and (2) this relationship would be more pronounced later in the season regardless of when parents initiated nests.

## 2 Materials and Methods

### 2.1 Study areas

We studied tree swallows breeding in nest boxes in 2008 and 2009 at two separate locations in Canada. The Saskatchewan site was located on the St. Denis National Research Area (SDNRA; 52^°^N, 106^°^W), 40 km east of the city of Saskatoon. This site had small groves of trees within an agricultural landscape composed of forage cover, agricultural crops, and numerous wetland basins. There were 165 boxes in 2008 and 160 in 2009 placed ~30 m apart and mounted on metal posts. Data were also collected in the vicinity of Prince George (53^°^N, 122^°^W), British Columbia (PG), at sites within an open agricultural habitat interspersed with small stands of deciduous and coniferous trees. In total, 123 (2008) and 139 (2009) nest boxes were placed ~30 m apart and mounted on wooden fence posts.

### 2.2 Nest monitoring and delay and brood size manipulations

Nest boxes were visited daily to determine clutch initiation and completion dates. When two females began laying eggs within 2 days of each other, each nest was randomly assigned to either the non‐delay (i.e., control) or delay group. On the morning the third egg was laid, all eggs were either picked up and immediately replaced in the nest (non‐delay) or collected and replaced with solid plastic eggs painted white (delay). Each subsequent egg in the laying sequence was handled (non‐delay) or collected and replaced with a dummy egg (delay) the morning they were laid. Collected eggs were transported to the laboratory and refrigerated at 8°C (Siikamäki, 1998). Clutch completion was determined when the clutch size remained the same for 3 days, at which time visits ceased (except delay nests). To mimic natural hatching asynchrony in delay nests, the first four eggs laid were returned 3 days following clutch completion and the remaining two to four eggs were returned the next day. Beginning 2 days prior to the estimated hatching date, all nests were visited daily to determine the date of first nestling emergence, hatching success, and brood size. The delay manipulation resulted in a hatching delay of ~5 days (control: mean incubation duration = 13.23 days, SD = 0.70, *n *=* *111; delay: mean incubation duration = 18.50 days, SD = 0.88, *n *=* *112) for both sites and years combined. Long‐term data from the SDNRA suggest that a 5‐day delay in breeding could result in a 13% decline in local recruitment of nestlings (Shutler et al., 2006).

We used a full factorial experimental design and crossed the delay treatment with a brood size manipulation. Three nests within the same delay treatment level that hatched on the same day were randomly assigned to brood enlargement, control, or reduction groups. When nestlings were two days old, they were weighed and two nestlings of intermediate mass (i.e., not the smallest or largest) were individually marked with non‐toxic marker and removed from reduction nests and placed in enlargement nests. Nestlings in control nests were weighed and returned to their own nest. This resulted in six manipulation combinations with similar final sample sizes of nests: non‐delay‐enlargement (*n *=* *34), non‐delay‐control (*n *=* *42), non‐delay‐reduction (*n *=* *35), delay‐enlargement (*n *=* *32), delay‐control (*n *=* *45), and delay‐reduction (*n *=* *35). Nestlings added to each enlarged brood originated from a single reduced brood with the exception of SDNRA in 2008 when embryo mortality occurred in some delay clutches (see also Wiggins, Pärt, & Gustafsson, 1998) and resulted in composite broods of nestlings from up to four nests to achieve intended brood sizes.

### 2.3 Adult and nestling measurements

When all viable eggs had hatched, parents were captured at the box, and mass (nearest 0.5 g measured with a spring scale) and length of the combined head and bill (“head‐bill”; nearest 0.01 mm with dial (SDNRA) or digital (PG) calipers) were recorded. To obtain an index of body condition for each bird, we used residuals of adult mass regressed against their head‐bill length and age of nestlings at capture (O'Brien & Dawson, 2013). Nestling age was calculated by denoting the presence of the first nestling as day 0 such that all nestlings in a box were assigned the same age. Individual nestlings were uniquely marked with nontoxic markers beginning on day 4 (or day 2 for swapped nestlings) and banded with aluminum bands at 12 (SDNRA) or 16 (PG) days of age. Measurements of mass (nearest 0.25 g using a spring scale) and length of the head‐bill (2008 only; as above for adults) were recorded for each nestling every other day from 4 to 16 days old. In 2009, length of head‐bill was measured when nestlings were 16 days old. Additionally, the length of the ninth primary flight feather was measured (nearest 0.5 mm with a ruler) every other day when nestlings were 8–16 days old. From these measurements, we calculated growth rate constants for each nestling. We fitted nonlinear logistic models (PROC NLIN; SAS Institute Inc., Cary, North Carolina, USA) for gain of body mass and growth of head‐bill using the Levenberg–Marquardt estimation method. For growth of ninth primary feathers, we fit a linear model (Dawson, Lawrie, & O'Brien, 2005). Residuals of nestling mass regressed against length of head‐bill at 16 days old were used as an index of nestling body condition.

### 2.4 Weather data and insect abundance

Ambient temperature (°C), rainfall (mm), and wind speed (ms^−1^) were recorded on each site. A daily weather index was calculated by summing standardized values (for both sites and years combined) of mean temperature, minimum temperature, total rainfall, mean wind speed, and maximum wind speed (Pelayo & Clark, 2003). Negative values indicated cool, wet, and windy weather conditions.

Passive insect samplers (see Quinney & Ankney, 1985, for design) were placed in the vicinity of nest boxes with the opening of the sampler 2 m above ground (*n *=* *2 and 4 at SDNRA in 2008 and 2009, respectively; *n *=* *2/year at PG). Insects accumulated in jars containing 70% ethanol and were collected and replaced after ~12 (SDNRA) or ~ 24 hr (PG) of sampling. Insects were stored in fresh ethanol until they could be dried and weighed (nearest 0.00001 g with an analytical balance; Harriman, Dawson, Clark, Fairhurst, & Bortolotti, 2014). Daily insect biomass was averaged among samplers on each site and corrected for sampling duration and mean wind speed during the sampling period (Quinney, Hussell, & Ankney, 1986). Mean weather index and mean insect biomass were calculated for each nest during the period when nestlings were 2–16 days old (i.e., for the duration of nestling measurements plus 2 days prior to the first nestling measurement).

### 2.5 Statistical analyses

To evaluate whether the extended incubation duration of the delay treatment affected adult body condition, we used generalized linear models (PROC GLM, normal distribution) to examine potential differences in adult condition at hatch among treatment groups and between sites and years. Eleven models were considered for each sex separately, including interactions among year, site, delay, and brood size manipulations.

Linear mixed effects models (PROC MIXED) were used to determine the influence of treatments (delay, brood size manipulation), clutch initiation date (standardized so that day 1 represented the first clutch initiation date each year at each site), insect biomass, weather index, condition index of female parents of the box where nestlings were raised in, site, and year (fixed effects) on nestling quality. Nestling quality was indexed by the following dependent variables: nestling mass, lengths of the head‐bill and primary feather at day 16, growth rates, and body condition. These analyses accounted for clustering of data by using nest identity of the box that nestlings were hatched in and raised in as random factors. Although brood size manipulations alter food available on a per capita basis (Berzins & Dawson, 2016; Bortolotti et al., 2011; Shutler et al., 2006), we could not directly manipulate the food intake of nestlings and thus included estimates of aerial insect biomass in analyses. Furthermore, we used generalized linear models (PROC GLM, normal distribution) to assess the relationship between mean insect biomass (when nestlings were 2–16 days old for each nest) and site, year, and date (based on the day the first nestling hatched for each nest and standardized so that day 1 represented the first hatching day each year at each site). We examined 11 a priori models. Food abundance is the main hypothesized mechanism underlying a seasonal deterioration of the environment, but weather conditions can alter the quality of nestling tree swallows independent of hatching date and parental quality (Dawson, 2008). Thus, we considered the effect of the weather index on nestling quality. Delay manipulations were intended to control for parental quality; that is, individuals that initiated breeding at the same time at the same study area were assumed to be of similar quality (Ardia, 2005; O'Brien & Dawson, 2013); however, parental quality may be an important predictor of nestling quality, independent of breeding time (Cichoń, Sendecka, & Gustafsson, 2006), so the index of adult body condition was included in analyses. Male body condition was not influential in any analysis and was removed from final analyses. To consider the ability of parents to compensate for raising broods larger than intended, we initially included the difference between manipulated brood size and intended brood size (i.e., brood size postmanipulation – clutch size) in analyses. This covariate was correlated with and did not outperform brood size manipulation in any model and thus was dropped from final analyses. Furthermore, the effect of female minimum age (minimum age determined by plumage characteristics and capture history) on nestling quality was considered in post hoc analyses. The inclusion of female minimum age in the most parsimonious model and as a single factor did not change the interpretation of our results. Clutch initiation date (*n *=* *222) was correlated with insect biomass (PROC CORR; *r* = −0.261, *P *<* *0.0001) and weather index (*r* = 0.296, *P *<* *0.0001); therefore, these covariates were not included in the same models. We considered 26 a priori models for each response variable (23 for growth of head‐bill), including interactions between treatments and clutch initiation date, and an intercept‐only (null) model. Parameters (β) were estimated using restricted maximum likelihood (PROC MIXED). A Kenward–Roger correction (Kenward & Roger, 1997) was used to calculate denominator degrees of freedom for linear mixed models. Predicted relationships between covariates and nestling quality relative to each hypothesis are shown in Table 1.

**Table 1:** Predicted effect or direction of relationships for the quality and date hypotheses between covariates and indices of quality for nestling tree swallows (*Tachycineta bicolor*)

Covariate	Quality	Date
Delay manipulation (delayed vs. non‐delayed)	o	−
Brood size enlargement		−
Brood size control		o
Brood size reduction		+
Insect biomass		+
Weather index		+
Clutch initiation date	−	−
Female body condition index	+	

A minus (−) symbol indicates a negative effect or direction, plus (+) signifies a positive effect or direction, (o) indicates no effect, and open boxes indicate that the covariate is not applicable to the hypothesis.

In addition to nestling quality, we evaluated the effect of treatments and the other variables described above on nestling fledging (fate) and recruitment. Due to the small numbers of nestlings that either died prior to fledging (67 of 1,353 nestlings) or recruited locally (43 of 1,286 fledglings), we examined nestling fate and local recruitment as binary responses by nest rather than by individual. The fate of each nest was classified as either all nestlings fledged or one or more nestlings died. Likewise, recruitment index was defined as one or more nestlings from a nest returned to the breeding population as an adult or none returned. We considered recaptures of marked nestlings until 2012, that is, 3–4 years following experiments, which was sufficient duration to detect >90% of all recruited offspring (*n *=* *530 (SDNRA, 1991–2012) and 137 (PG, 2002–2012) total recruits; unpublished data). Logistic regressions (PROC LOGISTIC) were used to test the effect of treatments, clutch initiation date, insect biomass, weather index, condition index of female parents of the box where nestlings were raised in, site, and year on survival to fledging and subsequent local recruitment of nestlings that fledged. We considered 26 a priori models for both response variables, including interactions between delay and brood size manipulations, manipulations and clutch initiation date, and an intercept‐only (null) model. The local recruitment index measures return rate and so should not be considered a surrogate for survival.

We used an information‐theoretic approach (Akaike's information criterion adjusted for sample size; AIC_c_) to examine the relative support for models within each candidate model set (Burnham & Anderson, 2002). The model with the smallest AIC_c_ value in each candidate set was considered the most parsimonious, but those within 2 AIC_c_ units of the best‐approximating model (ΔAIC_c_ < 2) were considered competitive (Burnham & Anderson, 2002). The estimates of the likelihood of the model relative to all models considered (Akaike weight; ω_i_) were also used to make inferences. We report β ± SE of the most parsimonious model unless stated otherwise. Sample sizes vary slightly among analyses due to missing measurements.

## 3 Results

### 3.1 Effects of treatments on parental condition

The best‐approximating model suggested that study site was the best predictor of female body condition and indicated that females at PG were in poorer condition than those at SDNRA (β = −0.738 ± 0.180; *n* = 221). Male body condition was poorer at PG (β = −1.148 ± 0.172; *n* = 215) and, although the top model also included year, this effect was negligible (β = −0.282 ± 0.152). There was no indication of an effect of delay treatment on female or male body condition. The top‐ranked model from analysis (PROC GLM) of mean mass of nestlings at day 4 was an additive model that included study site and delay treatment and indicated that nestling mass was lower at PG than SDNRA (β = −1.133 ± 0.386; *n* = 79) but did not differ by delay treatment (β = −0.243 ± 0.327).

### 3.2 Nestling size and growth

**Figure 1:**
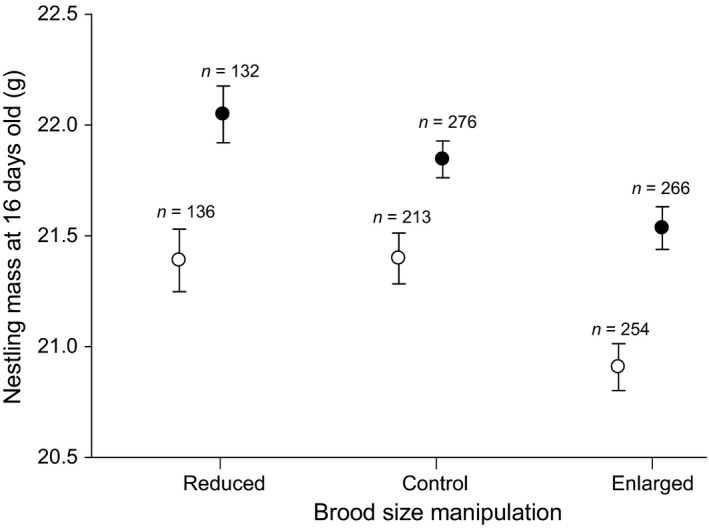
Mean (±SE) mass of 16‐day‐old nestling tree swallows (*Tachycineta bicolor*) raised during periods of low (open circles) and high (closed circles) levels of aerial insect biomass in control nests and those where brood size was reduced or enlarged by two nestlings at the St. Denis National Research Area, Saskatchewan, and Prince George, British Columbia, 2008–2009. Number of nestlings in each group is shown above error bars

Three models were competitive for describing body mass of 16‐day‐old nestlings (*n *=* *1277; Table 2). The best‐approximating model suggested that nestling mass increased with increasing insect biomass (β = 2.803 ± 0.496) and that nestlings in enlarged broods were lighter than those in control broods (β = −0.399 ± 0.163), but there was no difference in mass between nestlings in reduced and control broods (β = 0.148 ± 0.177). The second best model suggested an interaction between insect biomass and brood size manipulation. To explore this interaction, the relationship between brood size manipulation at low (≤median) and high (>median) levels of insect abundance was examined. Nestling mass was positively related to insect biomass at all levels of brood size manipulations; however, nestlings in enlarged broods were lighter than those in control broods (β = −0.552 ± 0.276, *n *=* *603) at low but not at high levels of insect abundance (β = −0.275 ± 0.206; *n *=* *674; Figure 1). Mass of nestlings in control and reduced broods did not differ at either level of insect biomass. Finally, the third‐ranked model indicated that nestlings in enlarged broods were lighter than their control counterparts (β = −0.389 ± 0.158), that delayed nestlings were lighter than those in non‐delayed nests (β = −0.529 ± 0.141), and that females in better body condition raised heavier nestlings (β = 0.286 ± 0.056).

**Table 2:** Model selection results for analyses that related size, growth, body condition, fate, and local recruitment of nestling tree swallows (*Tachycineta bicolor*) to measurements of parental and environmental quality and for an analysis examining correlates of mean insect biomass. Work was conducted at the St. Denis National Research Area, Saskatchewan, and near Prince George, British Columbia, 2008–2009. Only models with ΔAIC_c_ < 4 and the intercept‐only (null) model are presented with the exception of specific models of interest with precise parameter estimates

Response variable	Model structure^a^	*K* b	−2logL^c^	AIC_c_ d	ΔAIC_c_ e	ω*i* f
Mass (16 days old)	BroodManip + Insect	7	4525.52	4539.61	0.00	0.27
	BroodManip*Insect	9	4521.63	4539.77	0.16	0.25
	BroodManip + Delay + FCond	8	4523.96	4540.07	0.46	0.21
	FCond + BroodManip*Delay	10	4521.19	4541.36	1.75	0.11
	Global (BroodManip + Delay + FCond + CID + Year)	10	4521.49	4541.67	2.05	0.10
	Null	4	4563.27	4571.30	31.69	0.00
Length of head‐bill (16 days old)	Insect	5	2367.73	2377.78	0.00	0.97
	Null	4	2455.12	2463.15	85.37	0.00
Length of ninth primary feathers (16 days old)	BroodManip + Insect	7	7430.88	7444.97	0.00	0.45
	Insect	5	7435.24	7445.29	0.32	0.38
	Insect*CID	7	7433.08	7447.17	2.20	0.15
	Null	4	7467.19	7475.22	30.25	0.00
Mass growth rate constant	Null	4	−3131.22	−3123.19	0.00	0.96
Head‐bill growth rate constant	Null	4	−2267.44	−2259.36	0.00	0.88
	Insect	5	−2264.71	−2254.59	4.77	0.08
	Delay	5	−2262.82	−2252.71	6.65	0.03
Ninth primary feather growth rate constant	Insect	5	673.83	683.87	0.00	0.98
	Null	4	690.01	698.04	14.17	0.00
Nestling body condition	BroodManip + Delay + FCond	8	4250.05	4266.16	0.00	0.47
	BroodManip*Delay + FCond	10	4247.31	4267.49	1.33	0.24
	BroodManip + FCond	7	4253.73	4267.82	1.66	0.21
	Null	4	4284.37	4292.40	26.24	0.00
Fate^g^	Site	2	176.27	180.33	0.00	0.47
	BroodManip + Insect	4	173.89	182.08	1.75	0.20
	Site + Year	3	176.17	182.28	1.95	0.18
	Insect	2	179.12	183.18	2.85	0.11
	Null	1	209.022	211.04	30.71	0.00
Local recruitment^g^	Delay*CID	4	179.91	188.09	0.00	0.58
	Delay + Year	3	185.67	191.78	3.68	0.09
	Null	1	196.44	198.46	10.37	0.00
Insect biomass	Date + Site*Year	6	−329.29	−315.61	0.00	0.99
	Null	2	−205.03	−200.80	114.81	0.00

a Factors included delay treatment (Delay), brood size manipulation (BroodManip), mean insect biomass (Insect), clutch initiation date (CID), female body condition (FCond), Date, Site, Year, and an intercept‐only model (Null). Models with interactions (*) between factors also included the main effects.

b Number of estimable parameters.

c Deviance.

d Akaike's information criterion corrected for small sample size.

e Difference in AIC_c_ values between each model and the model with the lowest AICc value.

f Estimates of the likelihood of the model, given the data; normalized to sum to 1 (Burnham & Anderson, 2002).

g Summarized by nest.

Length of ninth primary feathers of 16‐day‐old nestlings was best described by two models (*n *=* *1300; Table 2), both indicating that feathers were longer when mean insect biomass was greater (model 1: β = 10.392 ± 1.841, model 2: β = 10.127 ± 1.837). The top‐ranked model also included brood size manipulation, but nestlings in enlarged (β = 0.010 ± 0.621) and reduced (β = 0.933 ± 0.654) broods did not differ from controls. Length of the head‐bill of 16‐day‐old nestlings (*n *=* *1299) was best described by the model with insect biomass (Table 2); nestlings raised during periods of greater insect biomass had longer head‐bills (β = 2.342 ± 0.228).

Variation in growth of nestling body mass (*n *=* *1273) was best explained by the null model. Growth rate of head‐bill (*n *=* *547) also was best explained by the null model, and models incorporating effects of insect biomass and delay treatment had weak support. Nestling head‐bill length grew faster with increasing insect biomass (β = 0.029 ± 0.015) and nestlings in delayed nests had slower rates of head‐bill growth than their non‐delayed counterparts (β = −0.011 ± 0.005). The growth of ninth primary feathers (*n *=* *1289) was best explained by an insect biomass model, with faster feather growth at higher levels of insect biomass (β = 0.640 ± 0.147; Table 2).

Nestling body condition (*n *=* *1275) was best explained by a model including delay treatment, brood size manipulation, and female body condition (Table 2). Nestlings in delayed nests were in poorer body condition than their non‐delayed counterparts (β = −0.323 ± 0.132) as were nestlings in enlarged broods relative to control (β = −0.360 ± 0.149) and reduced (β = −0.479 ± 0.146) broods, with no difference between nestlings in control and reduced broods (β = 0.119 ± 0.161). Females in better body condition raised nestlings in better condition (β = 0.283 ± 0.053).

### 3.3 Indices of nestling fate and recruitment

The best‐approximating model for nestling fate indicated that survival prior to fledging was lower at PG than SDNRA (β = −1.060 ± 0.192; *n *=* *221 nests). Also, nestling survival was positively associated with insect biomass (β = 6.750 ± 1.302) but lower for nestlings in enlarged broods relative to control broods (β = −0.612 ± 0.269).

**Figure 2:**
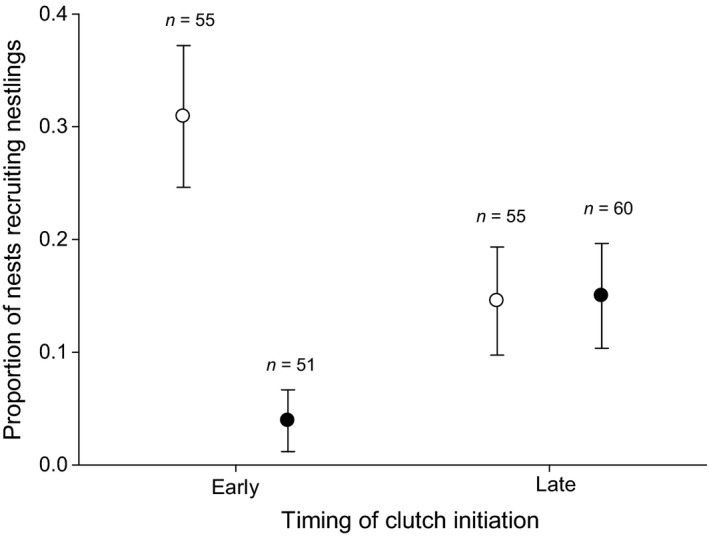
Proportion (±SE) of nests of tree swallows (*Tachycineta bicolor*) that recruited nestlings according to whether hatching was delayed (closed circles) or not (open circles), by timing of clutch initiation (early, late) at the St. Denis National Research Area, Saskatchewan, and Prince George, British Columbia, 2008–2009. Early timing represents nestlings produced by parents that initiated earlier than the median clutch initiation date, while late timing represents nestlings produced by parents that initiated on or after the median clutch initiation date. Number of nestlings in each group is shown above error bars

Of 221 nests included in analyses, one nestling per nest recruited from 30 nests, two nestlings per nest returned from five nests (all at SDNRA), and three from one nest at PG. All nests where more than one nestling recruited were non‐delayed nests and brood sizes had either been reduced or enlarged (i.e., no controls). Multiple recruits were produced from nests spanning a wide range of clutch initiation dates (range of standardized initiation dates = 3–10). Local recruitment index was best explained by an interaction between delay treatment and clutch initiation date. Recruitment index for nestlings in non‐delayed nests declined seasonally (β = −0.187 ± 0.106; *n *=* *110) but increased seasonally for nestlings in delayed nests (β = 0.247 ± 0.102; *n *=* *111; Figure 2). Furthermore, nestlings in non‐delayed nests were more likely to recruit locally than their delayed counterparts if they fledged from nests that were initiated before the median clutch initiation date (β = 1.197 ± 0.389; *n *=* *106), but this was not the case for late‐initiated nests (β = −0.018 ± 0.263; *n *=* *115; Figure 2). Although the pattern within early and late clutch initiation periods was the same between sites, the apparent seasonal increase in recruitment index for nestlings in delayed nests was driven by higher recruitment of this cohort at SDNRA as there was no difference in recruitment index of nestlings in delayed nests at PG regardless of timing of clutch initiation.

### 3.4 Insect biomass

Changes in mean insect biomass (when nestlings were 2–16 days old for each nest) were best explained by the model including date and an interaction between site and year (Table 2). To explore this interaction, sites were examined separately. Insect biomass decreased with later dates at both SDNRA (β = −0.020 ± 0.002; *n *=* *33) and PG (β = −0.008 ± 0.003; *n *=* *24) and was not different between years at SDNRA (β = 0.009 ± 0.020) but was lower in 2008 than 2009 at PG (β = −0.193 ± 0.021).

## 4 Discussion

**Figure 3:**
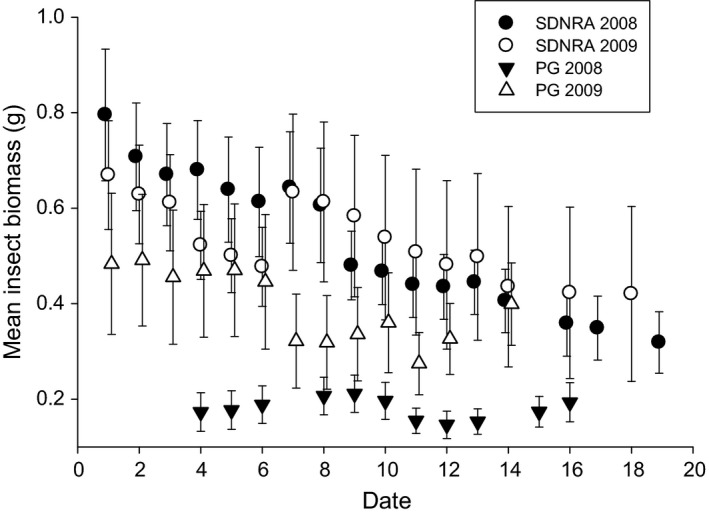
Mean (±SE) biomass of insects (averaged from when nestling tree swallows were 2–16 days old for each hatching day) in relation to date (day 1 represents the first day of nestling emergence at each site in each year) during the breeding season of tree swallows (*Tachycineta bicolor*) at the St. Denis National Research Area, Saskatchewan (SDNRA), and Prince George, British Columbia (PG), 2008–2009

We found that reduced reproductive success of late‐breeding tree swallows was causally related to a seasonal decline in environmental quality. Several indices of nestling quality were linked to insect biomass (mean biomass when nestlings were 2–16 days old for each nest), a resource that decreased with later hatching dates at both SDNRA and PG in both years (Figure 3). Other experimental tests which did not control for parental quality have documented support for the date hypothesis and have invoked indirect evidence of declining food abundance as a principle aspect of the deteriorating environment (Dawson, 2008; Norris, 1993; Öberg et al., 2013). Although our findings do not preclude potential additional effects, such as those of seasonally increasing parasite loads (but see Harriman et al., 2014) or some aspect of parental quality not accounted for by delaying hatch by 5 days, the seasonal deterioration of the environment was associated with declining food resources or less food per nestling, resulting in lower quality nestlings as the breeding season advanced.

There was little indication that early‐nesting parents raised better quality nestlings than did late‐nesters, although nestlings had higher body condition and were heavier at 16 days old when raised by females in better body condition. While these findings suggest that female quality is an important predictor of some indices of nestling quality, body condition of females did not decrease with clutch initiation date, and thus, these results are not consistent with predictions of the parental quality hypothesis. Nestlings in delayed nests had lower body condition and were lighter than their non‐delayed counterparts, possibly because parents with delayed clutches generally raised nestlings during periods of lower insect biomass.

Declining insect biomass was related to, and brood size enlargement resulted in, nestlings that were lighter, in lower body condition, had shorter head‐bills, shorter and slower growing ninth primary feathers, and were less likely to survive to fledge. These results imply fitness‐related costs of a seasonally diminishing food supply for late‐breeding individuals. Nestling tree swallows do not attain adult primary feather lengths while in the nest. Thus, late‐hatching nestlings may be doubly disadvantaged in that they grow shorter feathers in the nest due to poor food resources and then also have less time for feather growth prior to migration (O'Brien & Dawson, 2008). Furthermore, skeletal growth of tree swallows is often completed by day 16 (Wiggins, 1990), indicating that ontogenetic effects of food supply may be permanent (Potti & Merino, 1996). Finally, analyses of long‐term data indicate that body mass of nestling tree swallows is an important predictor of first‐year apparent survival, with heavier nestlings being more likely to recruit locally than lighter nestlings (Harriman et al., 2014; McCarty, 2001; Shutler et al., 2006).

We did not find that nestlings in reduced broods were of higher quality than controls despite evidence that nestlings in experimentally reduced broods receive more food per capita (Berzins & Dawson, 2016; Shutler et al., 2006). In this study, clutch sizes of parents attending reduced broods were slightly larger than those in control and enlarged broods, by chance alone, and this resulted in a smaller difference in brood sizes between reduced (mean = 4.16, SD = 0.90, *n* = 69) and control (mean = 5.93, SD = 1.22, *n* = 86) nests when compared with enlarged (mean = 8.30, SD = 0.86, *n* = 66) and control nests. This may explain why indices of nestling quality in reduced broods were not different from those in control broods.

Although brood size manipulations provided an experimental framework for testing food abundance as a mechanism of deteriorating environmental conditions, measurements of insect biomass provided an opportunity to examine seasonal variation in food supply and to address potential interactive effects of food supply and treatments. Indeed, we found some evidence that nestlings in enlarged broods were lighter than those in control and reduced broods, but only at low levels of insect biomass. These results indicate that the cost of raising enlarged broods depends on environmental conditions during the nestling period. Likewise, in a 6‐year brood size manipulation study of collared flycatchers (*Ficedula albicollis*), nestling condition was lower in experimentally enlarged broods and this effect was more pronounced in years with less food (Török, Hegyi, Tóth, & Könczey, 2004). Collectively, these findings underscore the importance of directly evaluating relationships between indices of environmental quality and fitness‐related traits, and annual fluctuations in these indices, when assessing the implications of late breeding.

Our results suggest that nestling survival was higher during periods of greater insect biomass. Furthermore, we detected a cost of enlarged brood sizes in the form of nestling mortality, independent of food abundance. Although enlarged broods may produce more fledglings (Shutler et al., 2006), this relationship may be affected by food supplies, with a greater number of fledglings produced from enlarged broods in food‐rich years and a threshold in the number of fledglings produced occurring in average food years (Török et al., 2004). Nestling survival was lower at PG than SDNRA possibly due to overall lower food supplies at PG (Figure 3); however, our work and a previous study (Harriman, 2014) suggest that parents at PG are in lower body condition than parents at SDNRA, which may also account for these results.

Interestingly, in 2008 at SDNRA and PG, feeding rates by parents did not vary with insect biomass (Bortolotti et al., 2011), suggesting that changes in parental provisioning rates did not contribute to observed declines in nestling quality with declining food supply. The difference in mean insect biomass between PG and SDNRA was greater in 2008 than 2009 (Figure 3), but despite these site differences, parental provisioning rates did not differ between SDNRA and PG in 2008 (Bortolotti et al., 2011). It is therefore unlikely that the relationship between insect biomass and nestling quality was driven by differential feeding rates in 2009 alone. However, short‐term measurements such as those conducted by Bortolotti et al. (2011) may not have been representative of the total parental provisioning effort during the entire nestling period, and subsequent work suggests that there may be a seasonal shift in the prey species provisioned to nestling swallows (Bortolotti, Clark, & Wassenaar, 2013). Although adult provisioning rates may be a good index of the amount of food delivered to broods by parents (McCarty, 2002), seasonal shifts in prey composition raise the possibilities of changing food quality or amounts being delivered to nestlings. Finally, insect abundance and parental provisioning behavior may be negatively affected by weather conditions such as rainfall (Tinbergen & Dietz, 1994) and cooler temperatures (Winkler, Luo, & Rakhimberdiev, 2013). Late‐hatched nestlings experienced greater amounts of rainfall than early‐hatched nestlings in 2008 (unpublished data) and parental provisioning rates were not measured during periods of rainfall (Bortolotti et al., 2011). Indeed, growth rates of tree swallow nestlings are slower during periods of rain and reduced availability of insects (McCarty, 2001; McCarty & Winkler, 1999b). Thus, parents of late‐hatching nestlings may be required to feed at greater rates during favorable weather conditions to fully compensate for frequent periods of interrupted or reduced provisioning.

Recruitment of offspring is generally higher when nestlings are raised during periods of greater food supply (Török et al., 2004). A fitness‐related cost of the delay treatment was reflected in reduced local recruitment of nestlings in delayed nests of early‐initiating parents when compared to their non‐delayed counterparts. Early‐initiating delayed parents actually produced fewer recruits than parents that initiated late. This may indicate that costs associated with early breeding are alleviated by environmental conditions during the nestling period. In particular, costs associated with egg formation are greater earlier in the season when food supplies are generally lower and temperatures are cooler (Nilsson, 1994; Schaper & Visser, 2013). Costs of early laying may be outweighed by the benefits of raising offspring when food is more abundant (i.e., when intended) for early‐breeding individuals. Thus, when these individuals are forced to raise offspring during periods of lower food abundance (i.e., later than intended), early‐breeding individuals are not able to compensate for these costs, subsequently passing them to nestlings. Our finding that recruitment was lower for individuals from delayed nests is similar to those who have used clutch removal and relaying (Verhulst et al., 1995) and cross‐fostering (Norris, 1993) manipulations to induce changes in timing of nestling rearing. In contrast, there was no difference in the likelihood of producing at least one recruit between late‐initiating delayed and non‐delayed parents. This is the only result that provides support for predictions of the quality hypothesis and indicates that effects of parental quality on offspring recruitment may be more important for late‐initiating individuals; however, it is important to note that the span of hatching dates was greater for late than early‐initiating parents due to the pattern in timing of clutch initiation (i.e., the distribution of clutch initiation dates is skewed to the right). Among early‐initiating parents, few delayed and non‐delayed clutches overlapped in hatching date (and thus environmental conditions), whereas a greater number of late‐initiating delayed and non‐delayed parents did and thus experienced similar environmental conditions. Furthermore, the local recruitment index measures return rate and does not take into account natal dispersal rates, which could have been greater for later‐hatched nestlings; however, past research on tree swallows suggests that this is unlikely (Winkler et al., 2005).

We invoke seasonally declining food resources as the mechanism for reduced reproductive success of late‐breeding tree swallows in this study because of the effect of experimental brood enlargement on nestling condition and mortality (which was more pronounced during periods of low insect biomass) and the observed decline in insect biomass during the nestling period. However, insect abundances sometimes remain steady or even increase during the breeding season (e.g., Dunn, Winkler, Whittingham, Hannon, & Robertson, 2011; McCarty & Winkler, 1999a; Nooker, Dunn, & Whittingham, 2005); thus, declining prey abundance is unlikely to be a universal driver of seasonally declining reproductive success. Insect biomass is an imperfect proxy for the food available to tree swallows because they are selective foragers so not all insects sampled represent equally suitable prey (McCarty & Winkler, 1999a). Furthermore, multiple studies have demonstrated (Nooker et al., 2005; Winkler & Allen, 1995) or invoked (Stutchbury & Robertson, 1988) the importance of female foraging efficiency to multiple measures of reproductive success. Although foraging efficiency can be viewed as a component of parental condition, inherent to individuals (and thus more so related to the quality hypothesis), environmental conditions could mediate foraging efficiency independent of individual quality. For example, we observed evidence of a shift in prey species to smaller, less desirable taxa provisioned to nestlings at SDNRA (Bortolotti et al., 2013) and there is a seasonal change in the selectivity of foraging tree swallows (McCarty, 1995); these patterns could be associated with lower foraging efficiency later in the breeding season. Seasonally declining foraging efficiency could manifest in years and sites without a seasonal change in total insect biomass, as long as there is a change in aerial insect community composition or foraging behavior. Thus, although we have invoked declining insect biomass as the cause of reduced reproductive success of late‐breeding individuals, other dimensions of food availability, unmeasured by this study, could be important.

The method of delaying hatch via egg storage provided the advantage of returning eggs to the female that laid them, which was particularly important in this context as egg quality, which has subsequent effects on offspring quality, varies with female quality and/or clutch initiation date (Ardia, Cooper, & Dhondt, 2006). However, like all experimental techniques employed to manipulate timing of breeding (see Verhulst & Nilsson, 2008), egg storage has disadvantages. By necessity, this technique resulted in extended incubation duration for delayed females, but we did not detect a cost of extended incubation on female (or male) body condition (see also Shutler et al., 2006; Wiggins et al., 1998; but see Wardrop & Ydenberg, 2003). Furthermore, Bortolotti et al. (2011) noted no difference between provisioning rates of delayed and non‐delayed parents irrespective of brood size. It is plausible that egg storage had a detrimental effect on embryo quality (Verhulst & Nilsson, 2008); however, we found no differences in nestling mass at 4 days of age in delayed and non‐delayed nests.

Timing of breeding is strongly associated with fitness (Daan & Tinbergen, 1997) and knowledge of what drives seasonal variation in fitness‐related components, such as reproductive success, is important for making inferences about consequences of altering breeding time on population dynamics, particularly in the context of landscape and climate changes (Both & Visser, 2001). Experimental tests of drivers of deteriorating environmental conditions are rare, despite the considerable attention this mechanism has received since Perrins's (1970) seminal paper. We accounted for parental quality and manipulated food availability per capita, while simultaneously considering indices of parental and environmental quality on two study sites over two breeding seasons. Our study provides strong evidence for the importance of food resources in mediating seasonal declines in avian offspring quality and survival. Thus, our findings have important implications for understanding the population dynamics of temperate‐breeding insectivorous birds, for which food availability is intricately linked to local weather conditions.

## Author Contributions

VBH, RGC, and RDD conceived and designed the study; VBH, LEB, RDD, and RGC collected data; VBH analyzed the data; VBH and LEB wrote the manuscript with input from all authors.

## Conflict of Interest

None declared.

## References

[ece32815-bib-0001] Anderson, J. H. , Faulds, P. L. , Atlas, W. I. , Pess, G. R. , & Quinn, T. P. (2010). Selection on breeding date and body size in colonizing coho salmon, *Oncorhynchus kisutch* . Molecular Ecology, 19(12), 2562–2573. 20492523 10.1111/j.1365-294X.2010.04652.x

[ece32815-bib-0002] Ardia, D. R. (2005). Individual quality mediates trade‐offs between reproductive effort and immune function in tree swallows. Journal of Animal Ecology, 74(3), 517–524.

[ece32815-bib-0003] Ardia, D. R. , Cooper, C. B. , & Dhondt, A. A. (2006). Warm temperatures lead to early onset of incubation, shorter incubation periods and greater hatching asynchrony in tree swallows *Tachycineta bicolor* at the extremes of their range. Journal of Avian Biology, 37(2), 137–142.

[ece32815-bib-0004] Berzins, L. L. , & Dawson, R. D. (2016). Experimentally altered plumage brightness of female tree swallows: A test of the differential allocation hypothesis. Behaviour, 153(5), 525–550.

[ece32815-bib-0005] Bortolotti, L. E. , Clark, R. G. , & Wassenaar, L. I. (2013). Hydrogen isotope variability in prairie wetland systems: Implications for studies of migratory connectivity. Ecological Applications, 23(1), 110–121. 23495640 10.1890/12-0232.1

[ece32815-bib-0006] Bortolotti, L. E. , Harriman, V. B. , Clark, R. G. , & Dawson, R. D. (2011). Can changes in provisioning by parent birds account for seasonally declining patterns of offspring recruitment? Canadian Journal of Zoology‐Revue Canadienne De Zoologie, 89(10), 921–928.

[ece32815-bib-0007] Both, C. , & Visser, M. E. (2001). Adjustment to climate change is constrained by arrival date in a long‐distance migrant bird. Nature, 411(6835), 296–298. 11357129 10.1038/35077063

[ece32815-bib-0008] Burger, C. , Belskii, E. , Eeva, T. , Laaksonen, T. , Magi, M. , Mand, R. , & Both, C. (2012). Climate change, breeding date and nestling diet: How temperature differentially affects seasonal changes in pied flycatcher diet depending on habitat variation. Journal of Animal Ecology, 81(4), 926–936. 22356622 10.1111/j.1365-2656.2012.01968.x

[ece32815-bib-0009] Burnham, K. P. , & Anderson, D. R. (2002). Model selection and multimodel inference: A practical information‐theoretic approach, second. New York: Springer‐Verlag.

[ece32815-bib-0010] Cichoń, M. , Sendecka, J. , & Gustafsson, L. (2006). Genetic and environmental variation in immune response of collared flycatcher nestlings. Journal of Evolutionary Biology, 19(5), 1701–1706. 16910999 10.1111/j.1420-9101.2006.01110.x

[ece32815-bib-0011] Daan, S. , & Tinbergen, J. M . 1997 Adaptation of life histories In Krebs J. R. & Davies N. B. (Eds.), Behavioural ecology, an evolutionary approach. 4th ed. (311–333). Oxford, UK: Oxford University Press.

[ece32815-bib-0012] Dawson, R. D. (2008). Timing of breeding and environmental factors as determinants of reproductive performance of tree swallows. Canadian Journal of Zoology‐Revue Canadienne De Zoologie, 86(8), 843–850.

[ece32815-bib-0013] Dawson, R. D. , Lawrie, C. C. , & O'Brien, E. L. (2005). The importance of microclimate variation in determining size, growth and survival of avian offspring: Experimental evidence from a cavity nesting passerine. Oecologia, 144(3), 499–507. 15891832 10.1007/s00442-005-0075-7

[ece32815-bib-0014] Dubiec, A. , & Cichoń, M. (2001). Seasonal decline in health status of Great Tit (*Parus major*) nestlings. Canadian Journal of Zoology‐Revue Canadienne De Zoologie, 79(10), 1829–1833.

[ece32815-bib-0015] Dunn, P. O. , Winkler, D. W. , Whittingham, L. A. , Hannon, S. J. , & Robertson, R. J. (2011). A test of the mismatch hypothesis: How is timing of reproduction related to food abundance in an aerial insectivore? Ecology, 92(2), 450–461. 21618924 10.1890/10-0478.1

[ece32815-bib-0016] Grüebler, M. U. , & Naef‐Daenzer, B. (2010). Fitness consequences of timing of breeding in birds: Date effects in the course of a reproductive episode. Journal of Avian Biology, 41(3), 282–291.

[ece32815-bib-0017] Gurney, K. E. B. , Clark, R. G. , & Slattery, S. M. (2012). Seasonal variation in pre‐fledging survival of lesser scaup *Aythya affinis*: Hatch date effects depend on maternal body mass. Journal of Avian Biology, 43(1), 68–78.

[ece32815-bib-0018] Harriman, V. B. (2014). Seasonal variation in quality and survival of nesting tree swallows (Tachycineta bicolor): tests of alternate hypotheses. PhD, University of Saskatchewan.

[ece32815-bib-0019] Harriman, V. B. , Dawson, R. D. , Clark, R. G. , Fairhurst, G. D. , & Bortolotti, G. R. (2014). Effects of ectoparasites on seasonal variation in quality of nestling tree swallows (*Tachycineta bicolor*). Canadian Journal of Zoology‐Revue Canadienne De Zoologie, 92(2), 87–96.

[ece32815-bib-0020] Kelly, M. G. , & Levin, D. A. (2000). Directional selection on initial flowering date in *Phlox drummondii (Polemoniaceae)* . American Journal of Botany, 87(3), 382–391. 10718999

[ece32815-bib-0021] Kenward, M. G. , & Roger, J. H. (1997). Small sample inference for fixed effects from restricted maximum likelihood. Biometrics, 53(3), 983–997. 9333350

[ece32815-bib-0022] Lyon, B. E. , Chaine, A. S. , & Winkler, D. W. (2008). Ecology ‐ A matter of timing. Science, 321(5892), 1051–1052. 18719273 10.1126/science.1159822

[ece32815-bib-0023] McCarty, J. P. (1995). Effects of short‐term changes in environmental conditions on the foraging ecology and reproductive success of Tree Swallows, Tachycineta bicolor. PhD, Cornell University.

[ece32815-bib-0024] McCarty, J. P. (2001). Variation in growth of nestling tree swallows across multiple temporal and spatial scales. Auk, 118(1), 176–190.

[ece32815-bib-0025] McCarty, J. P. (2002). The number of visits to the nest by parents is an accurate measure of food delivered to nestlings in Tree Swallows. Journal of Field Ornithology, 73(1), 9–14.

[ece32815-bib-0026] McCarty, J. P. , & Winkler, D. W. (1999a). Foraging ecology and diet selectivity of tree swallows feeding nestlings. Condor, 101(2), 246–254.

[ece32815-bib-0027] McCarty, J. P. , & Winkler, D. W. (1999b). Relative importance of environmental variables in determining the growth of nestling Tree Swallows *Tachycineta bicolor* . Ibis, 141(2), 286–296.

[ece32815-bib-0028] Nebel, S. , Mills, A. , McCracken, J. D. , & Taylor, P. D. (2010). Declines of aerial insectivores in North America follow a geographic gradient. Avian Conservation and Ecology, 5(2), 14.

[ece32815-bib-0029] Nilsson, J. A. (1994). Energetic bottle‐necks during breeding and the reproductive cost of being too early. Journal of Animal Ecology, 63(1), 200–208.

[ece32815-bib-0030] Nooker, J. K. , Dunn, P. O. , & Whittingham, L. A. (2005). Effects of food abundance, weather, and female condition on reproduction in tree swallows (*Tachycineta bicolor*). Auk, 122(4), 1225–1238.

[ece32815-bib-0031] Norris, K. (1993). Seasonal‐variation in the reproductive success of blue tits ‐ an experimental‐study. Journal of Animal Ecology, 62(2), 287–294.

[ece32815-bib-0032] Öberg, M. , Pärt, T. , Arlt, D. , Laugen, A. T. , & Low, M. (2013). Decomposing the seasonal fitness decline. Oecologia, 174(1), 139–150. 24013387 10.1007/s00442-013-2763-z

[ece32815-bib-0033] O'Brien, E. L. , & Dawson, R. D. (2008). Parasite‐mediated growth patterns and nutritional constraints in a cavity‐nesting bird. Journal of Animal Ecology, 77(1), 127–134. 18177333 10.1111/j.1365-2656.2007.01315.x

[ece32815-bib-0034] O'Brien, E. L. , & Dawson, R. D. (2013). Experimental dissociation of individual quality, food and timing of breeding effects on double‐brooding in a migratory songbird. Oecologia, 172(3), 689–699. 23229392 10.1007/s00442-012-2544-0

[ece32815-bib-0035] Pelayo, J. T. , & Clark, R. G. (2003). Consequences of egg size for offspring survival: A cross‐fostering experiment in ruddy ducks (*Oxyura jamaicensis*). Auk, 120(2), 384–393.

[ece32815-bib-0036] Perrins, C. M. (1970). Timing of birds breeding seasons. Ibis, 112(2), 242–255.

[ece32815-bib-0037] Potti, J. , & Merino, S. (1996). Parasites and the ontogeny of sexual size dimorphism in a passerine bird. Proceedings of the Royal Society B‐Biological Sciences, 263(1366), 9–12.

[ece32815-bib-0038] Price, T. , Kirkpatrick, M. , & Arnold, S. J. (1988). Directional selection and the evolution of breeding date in birds. Science, 240(4853), 798–799. 3363360 10.1126/science.3363360

[ece32815-bib-0039] Quinney, T. E. , & Ankney, C. D. (1985). Prey size selection by tree swallows. Auk, 102(2), 245–250.

[ece32815-bib-0040] Quinney, T. E. , Hussell, D. J. T. , & Ankney, C. D. (1986). Sources of variation in growth of tree swallows. Auk, 103(2), 389–400.

[ece32815-bib-0041] Reed, T. E. , Warzybok, P. , Wilson, A. J. , Bradley, R. W. , Wanless, S. , & Sydeman, W. J. (2009). Timing is everything: Flexible phenology and shifting selection in a colonial seabird. Journal of Animal Ecology, 78(2), 376–387. 19054224 10.1111/j.1365-2656.2008.01503.x

[ece32815-bib-0042] Saino, N. , Romano, M. , Ambrosini, R. , Rubolini, D. , Boncoraglio, G. , Caprioli, M. , & Romano, A. (2012). Longevity and lifetime reproductive success of barn swallow offspring are predicted by their hatching date and phenotypic quality. Journal of Animal Ecology, 81(5), 1004–1012. 22531043 10.1111/j.1365-2656.2012.01989.x

[ece32815-bib-0043] Schaper, S. V. , & Visser, M. E. (2013). Great tits provided with ad libitum food lay larger eggs when exposed to colder temperatures. Journal of Avian Biology, 44(3), 245–254.

[ece32815-bib-0044] Shutler, D. , Clark, R. G. , Fehr, C. , & Diamond, A. W. (2006). Time and recruitment costs as currencies in manipulation studies on the costs of reproduction. Ecology, 87(11), 2938–2946. 17168037 10.1890/0012-9658(2006)87[2938:tarcac]2.0.co;2

[ece32815-bib-0045] Shutler, D. , Hussell, D. J. T. , Norris, D. R. , Winkler, D. W. , Robertson, R. J. , Bonier, F. , ··· Stanback, M. T. (2012). Spatiotemporal patterns in nest box occupancy by tree swallows across North America. Avian Conservation and Ecology, 7(1), 3.

[ece32815-bib-0046] Siikamäki, P. (1998). Limitation of reproductive success by food availability and breeding time in pied flycatchers. Ecology, 79(5), 1789–1796.

[ece32815-bib-0047] Stutchbury, B. J. , & Robertson, R. J. (1988). Within‐season and age‐related patterns of reproductive‐performance in female tree swallows (*Tachycineta bicolor*). Canadian Journal of Zoology‐Revue Canadienne De Zoologie, 66(4), 827–834.

[ece32815-bib-0048] Tinbergen, J. M. , & Dietz, M. W. (1994). Parental energy‐expenditure during brood rearing in the great tit (*Parus major*) in relation to body‐mass, temperature, food availability and clutch size. Functional Ecology, 8(5), 563–572.

[ece32815-bib-0049] Török, J. , Hegyi, G. , Tóth, L. , & Könczey, R. (2004). Unpredictable food supply modifies costs of reproduction and hampers individual optimization. Oecologia, 141(3), 432–443. 15316767 10.1007/s00442-004-1667-3

[ece32815-bib-0050] Uller, T. , & Olsson, M. (2010). Offspring size and timing of hatching determine survival and reproductive output in a lizard. Oecologia, 162(3), 663–671. 19924446 10.1007/s00442-009-1503-x

[ece32815-bib-0051] Varpe, O. , Jørgensen, C. , Tarling, G. A. , & Fiksen, O. (2007). Early is better: Seasonal egg fitness and timing of reproduction in a zooplankton life‐history model. Oikos, 116(8), 1331–1342.

[ece32815-bib-0052] Verboven, N. , Tinbergen, J. M. , & Verhulst, S. (2001). Food, reproductive success and multiple breeding in the great tit *Parus major* . Ardea, 89(2), 387–406.

[ece32815-bib-0053] Verhulst, S. , & Nilsson, J. A. (2008). The timing of birds' breeding seasons: A review of experiments that manipulated timing of breeding. Philosophical Transactions of the Royal Society B‐Biological Sciences, 363(1490), 399–410. 10.1098/rstb.2007.2146 PMC2606757 17666390

[ece32815-bib-0054] Verhulst, S. , & Tinbergen, J. M. (1991). Experimental‐evidence for a causal relationship between timing and success of reproduction in the great tit *Parus m. major* . Journal of Animal Ecology, 60(1), 269–282.

[ece32815-bib-0055] Verhulst, S. , Vanbalen, J. H. , & Tinbergen, J. M. (1995). Seasonal decline in reproductive success of the great tit ‐ variation in time or quality. Ecology, 76(8), 2392–2403.

[ece32815-bib-0056] Wardrop, S. L. , & Ydenberg, R. C. (2003). Date and parental quality effects in the seasonal decline in reproductive performance of the Tree Swallow *Tachycineta bicolor*: Interpreting results in light of potential experimental bias. Ibis, 145(3), 439–447.

[ece32815-bib-0057] Wiggins, D. A. (1990). Clutch size, offspring quality, and female survival in tree swallow ‐ an experiment. Condor, 92(2), 534–537.

[ece32815-bib-0058] Wiggins, D. A. , Pärt, T. , & Gustafsson, L. (1998). Timing of breeding and reproductive costs in collared flycatchers. Auk, 115(4), 1063–1067.

[ece32815-bib-0059] Winkler, D. W. , & Allen, P. E. (1995). Effects of handicapping on female condition and reproduction in tree swallows (*Tachycineta bicolor*). Auk, 112(3), 737–747.

[ece32815-bib-0060] Winkler, D. W. , Luo, M. K. , & Rakhimberdiev, E. (2013). Temperature effects on food supply and chick mortality in tree swallows (*Tachycineta bicolor*). Oecologia, 173(1), 129–138. 23468236 10.1007/s00442-013-2605-z PMC3751296

[ece32815-bib-0061] Winkler, D. W. , Ringelman, K. M. , Dunn, P. O. , Whittingham, L. A. , Hussell, D. J. T. , Clark, R. G. , & Ardia, D. (2014). Latitudinal variation in clutch size‐lay date regressions in *Tachycineta* swallows: Effects of food supply or demography? Ecography, 37(7), 670–678.

[ece32815-bib-0062] Winkler, D. W. , Wrege, P. H. , Allen, P. E. , Kast, T. L. , Senesac, P. , Wasson, M. F. , & Sullivan, P. J. (2005). The natal dispersal of tree swallows in a continuous mainland environment. Journal of Animal Ecology, 74(6), 1080–1090.

